# Chemical analysis and evaluation of antioxidant and antimicrobial activities of fruit fractions of *Mauritia flexuosa* L. f. (Arecaceae)

**DOI:** 10.7717/peerj.5991

**Published:** 2018-11-28

**Authors:** Carla de Fatima Alves Nonato, Débora Odília Duarte Leite, Rafael Caldas Pereira, Aline Augusti Boligon, Jaime Ribeiro-Filho, Fabíola Fernandes G. Rodrigues, José Galberto M. da Costa

**Affiliations:** 1 PostGraduate Program in Ethnobiology and Nature Conservation, Universidade Regional do Cariri, Crato, Ceará, Brazil; 2 Department of Biological Chemistry, Universidade Regional do Cariri, Crato, Ceará, Brazil; 3 PostGraduate Program in Pharmaceutical Sciences, Universidade Federal de Santa Maria, Santa Maria, Rio Grande do Sul, Brazil; 4 University Center UniLeão, Juazeiro do Norte, Ceará, Brazil

**Keywords:** *Mauritia flexuosa*, Phenolic compounds, ABTS, Chelation, Antimicrobial, Antibiotic modulation

## Abstract

**Background:**

Fruit consumption is currently considered beyond the nutritional aspects because of the important roles in disease prevention and benefits to health. The objective of this study was to characterize the chemical profile and evaluate the antioxidant and antimicrobial properties of different fractions obtained from fruit pulps of *Mauritia flexuosa* (MFFs).

**Methods:**

Initially, chloroform, ethyl acetate and ethanol fractions were obtained from the pulps. Quantifications of total phenols and flavonoids were performed using the methods of Folin-Ciocalteu and complexation with aluminum chloride, respectively. Standard samples were used to identify and quantify phenolic acids and flavonoids using high-performance liquid chromatography with a diode-array detector (HPLC-DAD). The antioxidant capacity of the fractions was verified by sequestration of the free radical 2,2′-azino-bis (3-ethylbenzothiazoline-6-sulphonic acid (ABTS) and iron chelating activity. The antimicrobial activity was determined using the microdilution method and the modulating activity was determined using sub-inhibitory concentrations of the fractions in association with antibiotics.

**Results:**

The chemical analyzes revealed the presence of catechin, caffeic acid, rutin, orientin, quercetin, apigenin, luteolin and kaempferol, where all are present in the ethyl acetate fraction. The fractions exhibited moderate antioxidant and antimicrobial activities against Gram-positive and *Candida* strains in addition to modulating the activity of conventional antibiotics. The most expressive result was obtained from the association of the chloroform fraction with cefotaxime, which produced a synergistic effect, reducing the minimum inhibitory concentration (MIC) of the antibiotic from 1,024 to 256 μg/mL.

**Discussion:**

The fractions presented a constitution rich in phenolic compounds, especially flavonoids. The data obtained demonstrated that the fractions presented moderate antioxidant activity by acting both as primary and secondary antioxidants. The fractions presented antimicrobial and antibiotic potentiating activities, being the first record of modulating effect of fractions of this species against the studied microbial strains, but failed in modulating the activity of antifungal drugs, indicating that this plant has the potential to be used in the development of therapeutic alternatives against resistant bacteria. The constitution phenolic the fractions may be responsible for their pharmacological properties in vitro.

## Introduction

Over the past decade, epidemiological studies have indicated the importance of fruit and vegetable consumption for health, including for the prevention of chronic noncommunicable diseases (Costa, Vasconcelos & Corso, 2012). Functional foods are those that, besides dietary functions, have metabolic and physiological effects in the organism (Braga & Barleta, 2017).

The protective effect of fruits and vegetables against chronic degenerative diseases has been attributed to the presence of several antioxidant substances, including vitamins such as ascorbic acid, α-tocopherol and β-carotene. However, phenolic compounds are the main phytochemicals present in foods with antioxidant action (Garcia-Salas et al., 2010; Sucupira et al., 2014). Beyond to the antioxidant activity, polyphenols are recognized for their antimicrobial activity, because the chemical structure of the compounds facilitate their interaction with several intracellular targets, which favors the microbicidal effect (Cushnie & Lamb, 2005; Davidson, Sofos & Branen, 2005).

*Mauritia flexuosa* L. f. (Arecaceae) is a plant species that is popularly known as “buriti”. This plant stands out among the Brazilian species because of its wide use both in food and for medicinal purposes. Because of the typical aroma and peculiar flavor, the fruits are consumed either *in natura* or processed in the forms of juices, sweets, oil and ice creams (Lorenzi et al., 2004; Sampaio, 2011). Previous studies have demonstrated that *M. flexuosa* is rich in bioactive compounds such as carotenoids, tocopherols and phenolic compounds, besides presenting various biological actions such as chemoprotective, photoprotective against UVA and UVB radiation, antithrombotic, hypolipidemic, hypoglycemic and cicatrizing (Bataglion et al., 2014; Cândido, Silva & Agostini-Costa, 2015; Freire et al., 2016).

Therefore, in view of the importance of this species and its biological potential, the objective of this study was to characterize the chemical profile and evaluate the antioxidant and antimicrobial properties of different fractions obtained from fruit pulps of *Mauritia flexuosa*, being the first record of modulating effect of fractions of this species against the studied microbial strains.

## Materials and Methods

### Plant material

The fruits of *M. flexuosa* L. f. were collected in the Environmental Protection Area—APA -Chapada do Araripe (7°15′33.37″S 39°28′6.95″O) in October 2016, in the municipality of Crato, Ceará, Brazil. A voucher specimen was deposited in the “Herbário Caririense Dárdano de Andrade-Lima—HCDAL” at the Regional University of Cariri—URCA under register number 12.620. Authorization to collect botanical material was provided by the Authorization and Biodiversity Information System (SISBIO) of the Chico Mendes Institute for Biodiversity Conservation (ICMBio), registered under No. 61003-1.

### Drugs, reagents and equipment

Only analytical grade chemicals were used in this study. Acetonitrile, ethyl alcohol, formic acid, catechin, Folin-Ciocalteu reagent and gallic acid were purchased from Merck (Darmstadt, Germany). Apigenin, rutin, orientin, luteolin, kaempferol, quercetin, 2,2′-Azino-bis (3-ethylbenzothiazoline-6-sulfonic acid (ABTS), Trolox, 2, 4, 6-Tri(2-pyridyl)-s-triazine (TPTZ), phenanthroline, amikacin, gentamicin, benzylpenicillin, cefotaxime, fluconazole and ketoconazole were purchased from Sigma Chemical Co. (St. Louis, MO, USA). The antibiotics were dissolved in sterile water. High performance liquid chromatography (HPLC-DAD) was performed with a Shimadzu Prominence Auto Sampler (SIL-20A) HPLC system (Shimadzu, Kyoto, Japan), equipped with Shimadzu LC-20AT with reciprocating pumps connected to a DGU 20A5 degasser with a CBM 20A integrator, SPD-M20A diode array detector and LC solution 1.22 SP1 software.

### Preparation of the fractions

The chloroform (FCB), ethyl acetate (FAB) and ethanolic (FEB) fractions were obtained in a Soxhlet apparatus. About 800 g of the fruit pulp were subjected to extractions of 6 to 8 h in each solvent, with solvent exchange in the order of polarity and after complete evaporation. Then, the solutions were concentrated on rotary evaporator (Model 801, Fisatom, Brazil) at 50 °C under reduced pressure, yielding 1.34%, 0.87% and 2.9% (w/w) of FCB, FAB and FEB, respectively.

### Chemical analysis

#### Qualitative Chemical Prospecting

The identification of the classes of secondary metabolites in the fractions were performed according to the methodology of proposed by Matos (1997), in which the fractions were subjected to screening for the identification of the major classes of secondary metabolites, through chemical reaction resulting in development color and/or precipitate, characteristic for each metabolite class.

#### Determination of total phenols and flavonoids

The determination of phenols followed the method of Singleton, Orthofer & Lamuela-Raventós (1999). Briefly, dilutions of the fractions were oxidized by the Folin-Ciocalteu reagent and neutralized with sodium carbonate. The concentrations used ranged from 0.05 to 5.0 µg/mL. The readings were performed at 765 nm in a spectrophotometer and the analysis were performed in triplicate. The content of compounds was calculated from the calibration curve using gallic acid (AG), and the results were expressed in µg eq.AG/g.

The quantification of flavonoids was performed according to the methodology described by Kosalec et al. (2004) with adaptations. Concentrations ranging from 1 to 20 µg/mL were used. The tubes containing the samples were added with 750 µL of methanol, 40 µL of 10% aluminum chloride, 40 µL of potassium acetate and 1,120 µL of water. The readings were performed in a spectrophotometer at 415 nm and the analysis were made in triplicate.

#### Quantification of phenolic compounds and flavonoids by HPLC-DAD

*Mauritia flexuosa* fractions at a concentration of 1.2 mg/mL were injected using a SIL-20A Shimadzu Auto sampler. Separations were carried out using a Phenomenex C18 column (4.6 mm × 250 mm ×5 µm particle size). The mobile phase comprised solvent A [water: formic acid (98:2, v/v)] and solvent B (acetonitrile), at a flow rate of 0.6 mL/min and injection volume of 40 µL. The gradient program was started with 95% of A and 5% of B until 2 min and changed to obtain 25%, 40%, 50%, 70%, and 80% B at 10, 20, 30, 50, and 70 min, respectively, following the method described by Irondi et al. (2017), with slight modifications. The samples and mobile phase were filtered through a 0.45 µm membrane filter (Millipore, Burlington, MA, USA) and degassed by ultrasonic bath prior to use. Stock solutions of reference standards were prepared at concentrations ranging from 0.030 to 0.500 mg/mL. Quantifications were carried out by integration of the peaks using the external standard method, at 280 nm for catechin; 327 nm for caffeic acid; and 366 for apigenin, luteolin, rutin, quercetin, orientin and kaempferol. The chromatography peaks were confirmed by comparing its retention time with those of reference standards and by DAD spectra (200 to 600 nm). All chromatography operations were carried out at ambient temperature and in triplicate.

Limit of detection (LOD) and limit of quantification (LOQ) were calculated based on the standard deviation of the responses and the slope using three independent analytical curves, as defined by Sabir et al. (2012). LOD and LOQ were calculated as 3.3 and 10 *σ*∕*S*, respectively, where *σ* is the standard deviation of the response and *S* is the slope of the calibration curve.

### Evaluation of the antioxidant activity

#### Capture of the ABTS free radical

As previously, the fractions were used at concentrations ranging from 14 to 1,400 µg/mL. Under the light, a 30 µL aliquot of each concentration was transferred test tubes with 3.0 mL of the ABTS radical. The readings were performed 6 min after the reaction using a spectrophotometer with a wavelength of 734 nm. Trolox was used as positive control and methanol was used as a white (Rufino et al., 2007). To obtain the total antioxidant activity, the equivalent to 1,000 µM of the Trolox standard was substituted in the straight-line equation of the absorbance plot.

#### Fe^2+^ chelating activity

The chelation capacity of the fractions was measured according to the method proposed by Puntel, Nogueira & Rocha (2005) with adaptations. A volume of 100 µL of each concentration (14–1,400 µg/mL) was added with 300 µL of a FeSO_4_ solution (2 mM) and 336 µL 1.0 M TRIS-HCl (pH 7.4). The solutions were incubated in the dark for 5 min. Then, 26 µL of phenanthroline (0.25%) was added. The readings were performed at 510 nm using a spectrophotometer. A solution prepared without addition of the sample in the absence of incubation was used as a blank.

### Antimicrobial assays

#### Determination of the Minimum Inhibitory Concentration (MIC)

The antimicrobial activity of the fractions was evaluated by the microdilution method, using the document M07-A10 as a reference (CLSI, 2015). The assay was performed with 4 bacterial (*Bacillus cereus* INCQS 00303, *Staphylococcus aureus* ATCC 25923, *Escherichia coli* ATCC 25922 and *Salmonella choleraesuis* 00038 INCQS) and 3 yeast (*Candida albicans* INCQS 40006, *Candida krusei* 40095 INCQS and *Candida tropicalis* INCQS 40042) strains.

The fractions were diluted in sterile distilled water and dimethyl sulfoxide (1,024 µg/mL). Serial dilutions were performed to obtain concentrations ranging from 512 to 8 µg/mL. The tests were performed in triplicate and the plates were incubated at 35 ± 2 °C for 24 h. The results were analyzed by colorimetric reaction after addition of 25 µL of a solution of resazurin (0.01%) to each well after incubation. The minimum inhibitory concentration (MIC) was defined as the lowest extract concentration capable of inhibiting the growth of microorganisms.

### Evaluation of the modulating effect of MFFs in association with antimicrobial drugs

The modulating effect of the MFFs was analyzed by combining them with aminoglycosides (amikacin and gentamicin), beta-lactams (benzylpenicillin and cefotaxime) and azoles (fluconazole and ketoconazole) according to the methodology proposed by Coutinho et al. (2008). The bacterial strains *Bacillus cereus* INCQS 00303 and *Salmonella choleraesuis* INCQS 00038, and the fungal strains *Candida albicans* INCQS 40006, *Candida krusei* INCQS 40095 and *Candida tropicalis* INCQS 40042 were the microorganisms used. The MFFs were used at sub-inhibitory concentrations (MIC/8) obtained in specific culture medium (10%). The solutions were distributed in microdilution plates followed by addition of different concentrations of the antimicrobials obtained by serial dilution from an initial solution of 1,024 µg/mL. In the control, antibiotics were not added to the strains. The plates were incubated at 35 ± 2 °C for 24 h and read by colorimetry by the addition of 25 µL of resazurin solution (0.01%).

### Statistical analysis

Differences between groups of HPLC and antioxidant assays results were assessed by an analysis of variance model and Tukey’s test. The microbiological results were expressed as geometric mean and analysis of results was applied to two-way ANOVA followed by Bonferroni posttests using GraphPad Prism 6.0 software. Results with *P* < 0.05 were considered as statistically significant.

## Results

### Chemical analysis

#### Qualitative chemical prospecting

The chemical prospection of the fractions revealed the presence of several classes of secondary metabolites phenolics (Table 1), especially flavonoids.

**Table 1:** Classes of secondary metabolites identified in the fractions obtained from the fruit pulps of *M. flexuosa*. 1: Phenols; 2: Pyrogallic tannins; 3: Condensed tannins; 4: Anthocyanins; 5: Anthocyanidins; 6: Flavones; 7: Flavonols; 8: Xanthones; 9: Chalcones; 10: Aurones; 11: Flavononols; 12: Leucoantocyanidins; 13: Catechins; 14: Flavonones; 15: Alkaloids. (+) present; (−) absent.

**Fractions**	**Class of Secondary Metabolites**														
	1	2	3	4	5	6	7	8	9	10	11	12	13	14	15
FCB	–	–	–	–	–	+	+	+	+	+	+	+	+	+	–
FAB	–	–	–	–	–	+	+	+	+	+	+	+	+	+	–
FEB	–	–	–	–	–	+	+	+	+	+	+	+	+	+	–

#### Determination of total phenols and flavonoids

The content of flavonoids in the chloroform fraction was approximately twice less than in the ethyl acetate and ethanolic fractions, demonstrating that there are significant differences in the flavonoid content of the chloroform fraction and the other fractions. However, there were no differences between the flavonoid content of the ethanolic and ethyl acetate fractions. Regarding the content of phenols, the fractions presented slightly differences, as shown in Table 2.

**Table 2:** Content of total phenols and flavonoids in the fractions obtained from the fruit pulps of *M. flexuosa*. These results are expressed as mean ± SD (*n* = 3). The averages followed by different letters differ by Tukey’s test with a *p* < 0.05.

Fractions	Phenols (µg eq.AG/g)	**Flavonoids** (µg eq.Q/g)
**FCB**	22.51 ± 0.31a	5.99 ± 0.07a
**FAB**	26.84 ± 0.09b	11.82 ± 0.02b
**FEB**	22.69 ± 0.04a	12.47 ± 0.00b

#### Quantification of phenolic compounds and flavonoids by HPLC-DAD

**Figure 1:**
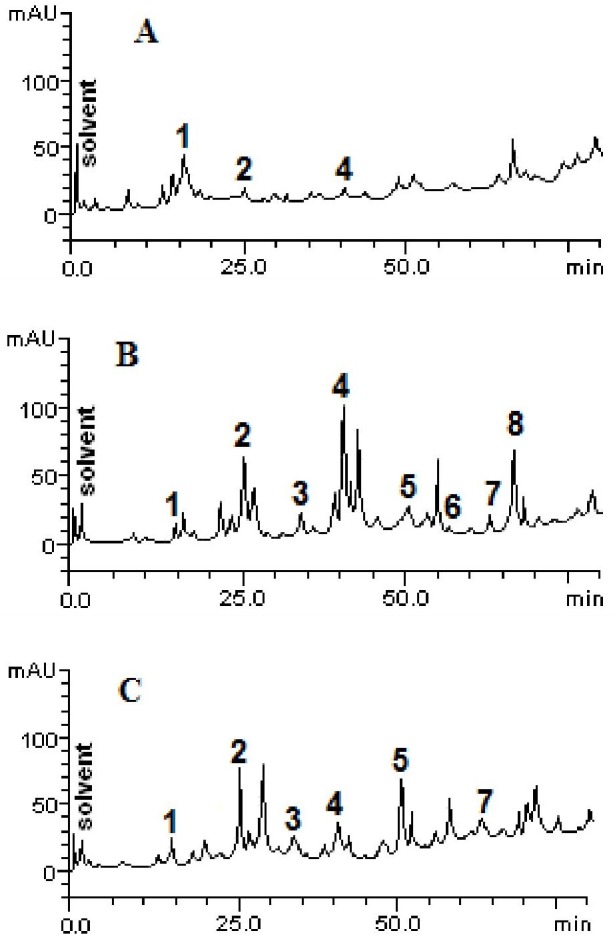
High performance liquid chromatography profile of chloroform (A), ethyl acetate (B) and ethanolic (C) fractions of *Mauritia flexuosa* fruit pulps. Representing the following compounds: Catechin (peak 1), caffeic acid (peak 2), rutin (peak 3), orientin (peak 4), quercetin (peak 5), apigenin (peak 6), luteolin (peak 7) and kaempferol (peak 8).

**Figure 2:**
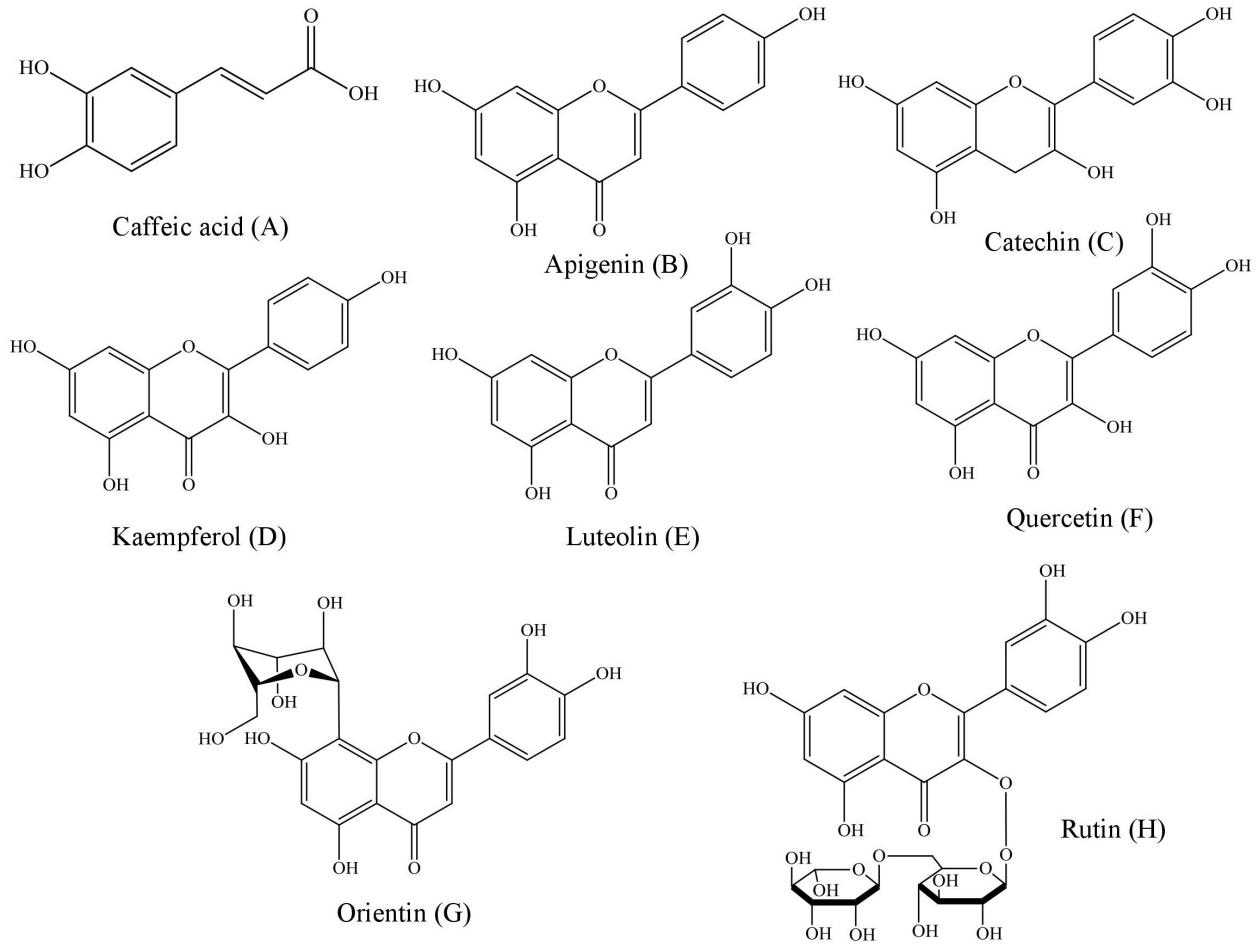
Structural representations of the phenolic compounds detected in the fruit pulp fractions of *Mauritia flexuosa*. (A) Caffeis acid, (B) apigenin, (C) catechin, (D) kaempferol, (E) luteolin, (F) quercetin, (G) orientin, (H) rutin.

The chromatographic profiles of the fractions from HPLC analyzes are shown in Fig. 1. Samples contain other compounds in smaller amounts, besides catechin (retention time -tR = 14.27 min, peak 1), caffeic acid (tR = 24.93 min, peak 2), rutin (tR = 41.07 min, peak 4), quercetin (tR = 52.09 min, peak 5), apigenin (tR = 56.38 min, peak 6), luteolin (tR = 64.11 min, peak 7) and kaempferol (tR = 67.02 min, peak 8). The HPLC analysis demonstrated variability between the total and individual values of each compound (Fig. 2), according to each fraction.

The results showed that the main phenolic compounds identified in the samples were orientin (5.29 ± 0.01–0.13 ± 0.02 mg/g), caffeic acid (3.96 ± 0.03–0.16 ± 0.01 mg/g) and quercetin (3.87 ± 0.05–1.84 ± 0.05 mg/g). In the ethyl acetate fraction, it was possible to quantify all the standards used in the analysis, whereas in the ethanolic fraction, apigenin and kaempferol were not identified, and in the chloroform fraction only catechin, caffeic acid and orientin were identified (Table 3).

**Table 3:** Components of fruit pulp fractions of *Mauritia flexuosa*. These results are expressed as mean ± SD (*n* = 3). The averages followed by different letters differ by Tukey’s test with *p* < 0.05, for the compounds of the same fraction.

**Compounds**	**FCB (mg/g)**	**FAB (mg/g)**	**FEB (mg/g)**	**LOQ (µg/mL)**	**LOD (µg/mL)**
Catechin	2.27 ± 0.04a	0.35 ± 0.01c	1.43 ± 0.02b	0.015	0.042
Caffeic acid	0.16 ± 0.01b	3.72 ± 0.04a	3.96 ± 0.03a	0.008	0.023
Rutin	–	1.46 ± 0.03a	1.71 ± 0.01a	0.023	0.034
Orientin	0.13 ± 0.02c	5.29 ± 0.01a	1.85 ± 0.01b	0.032	0.051
Quercetin	–	1.84 ± 0.05b	3.87 ± 0.05a	0.025	0.075
Apigenin	–	0.15 ± 0.02a	–	0.011	0.049
Luteolin	–	0.42 ± 0.02b	1.47 ± 0.04a	0.019	0.022
Kaempferol	–	3.69 ± 0.01a	–	0.003	0.029

As can be seen, the content of caffeic acid was similar in the ethyl acetate and ethanolic fractions. However, the chloroform fraction had a more than 20-fold lower content of the compound. Significant amounts of orientin were found in the ethyl acetate fraction, but in the other fractions, the content of this compound was significantly lower. For quercetin, significantly different values were found between the ethanolic and ethyl acetate fractions (3.87 ± 0.05 mg/g and 1.84 ± 0.05 mg/g, respectively). However, this compound was not quantified in the chloroform fraction and kaempferol was identified only in the ethyl acetate fraction.

### Antioxidant assays

#### Capture of the ABTS free radical

The effect of the fraction in the capture of the ABTS radical is shown is shown in Table 4. The fractions presented significantly differences in the capacity to capture the ABTS radical. The FCB presented the strongest activity, followed by FEB and FAB.

#### Fe^2+^ chelating activity

**Figure 3:**
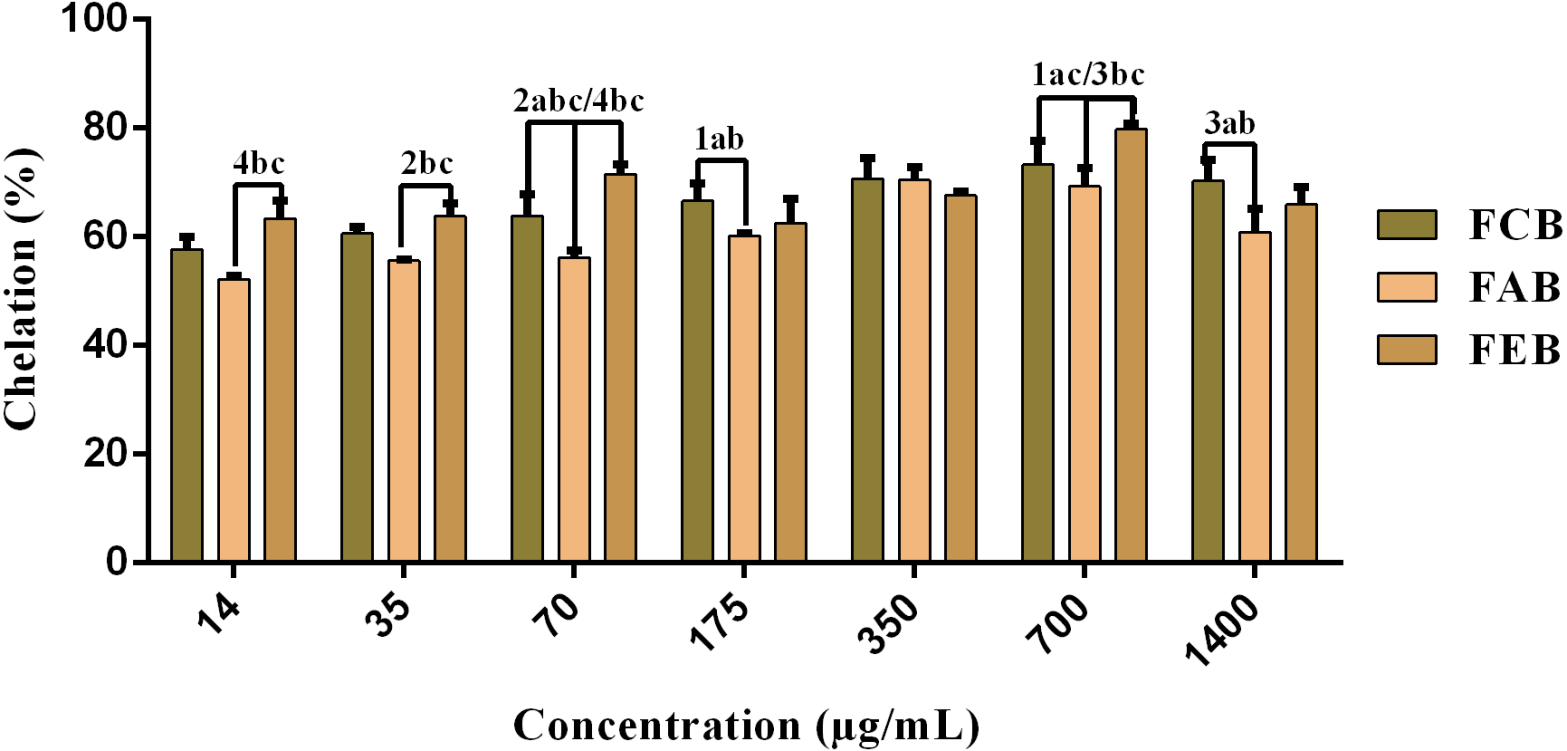
Iron chelating activity of the fruit pulp fractions obtained from *M. flexuosa*. Values are expressed as mean ±SD (*n* = 3). FCB (A), FAB (B) and FEB (C). The numbers 1–4 express the significance in the fractions in the same concentration. 1: *p* < 0.05, 2: *p* < 0.01, 3: *p* < 0.001 e 4: *p* < 0.0001. The data were analyzed by ANOVA and Tukey’s test.

The fractions studied in this work presented elevated chelating activity, whose response was concentration dependent (14–700 µg/mL) and presented significant differences when considering the same concentrations of the different samples (Fig. 3). However, considering the maximum chelation percentage, there were no significant variations, with a maximum percentage of 78.2% for FCB, 72.9% for FAB and 80.9% for FEB.

**Table 4:** Antioxidant activity values of fruit pulp fractions of *M. flexuosa*, according to the ABTS free radical capture. These results are expressed as mean ± SD (*n* = 3). The averages followed by different letters differ by Tukey’s test with a *p* < 0.05.

**Samples**	AA (μM trolox/g)
FCB	83.17 ± 3.23a
FAB	23.39 ± 0.10b
FEB	43.65 ± 0.12c

### Antimicrobial assays

#### Determination of the minimum inhibitory concentration (MIC)

The values of the minimal inhibitory concentrations obtained from the tested strains are shown in Table 5.

**Table 5:** Minimum inhibitory concentration of fruit pulp fractions of *M. flexuosa* against bacterial and fungal strains.

**Microorganisms**	**MIC (µg/mL)**		
	**FCB**	**FAB**	**FEB**
*Bacillus cereus* INCQS 00303	≥1,024	426.66	426.66
*Staphylococcus aureus* ATCC 25923	≥1,024	≥1,024	≥1,024
*Escherichia coli* ATCC 25922	≥1,024	≥1,024	≥1,024
*Salmonella choleraesuis* INCQS 00038	≥1,024	≥1,024	≥1,024
*Candida albicans* INCQS 40006	512	512	512
*Candida krusei* INCQS 40095	512	512	512
*Candida tropicalis* INCQS 40042	512	512	512

**Notes.**

ATCC American Type Culture Collection INCQS National Institute for Quality Control in Health

#### Modulating activity by direct contact

**Figure 4:**
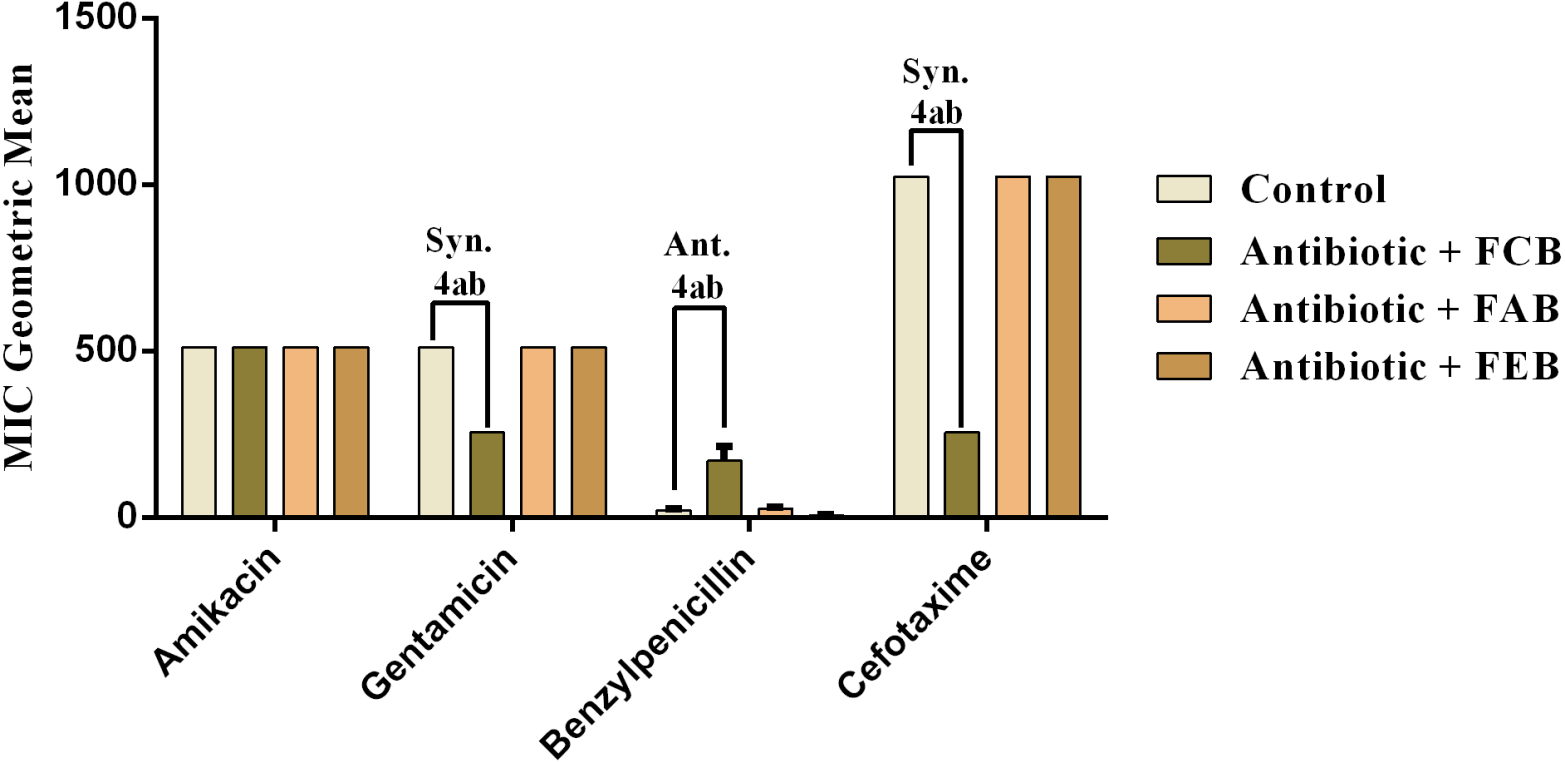
Modulating effect of FCB (B), FAB (C) and FEB (D) on the antibiotic activity of amikacin, gentamicin, benzylpenicillin and cefotaxime against the *Bacillus cereus* (INCQS 00303) strain. Control (A). The numbers 1–4 express the significance of the association between fraction + antibiotic. 1: *p* < 0.05, 2: *p* < 0.01, 3: *p* < 0.001 e 4: *p* < 0.0001. Data were analyzed by ANOVA and Bonferroni’s test.

**Figure 5:**
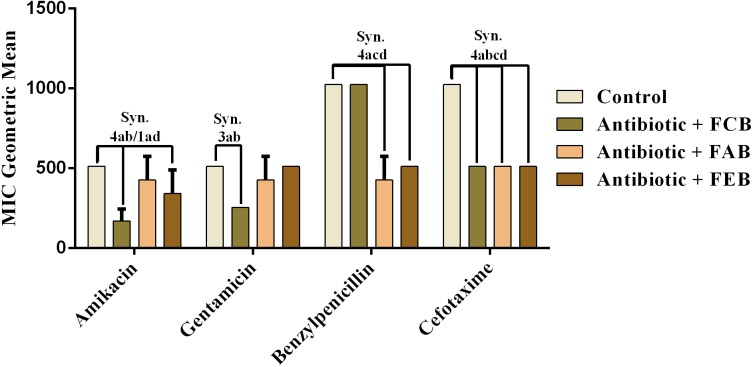
Modulating effect of FCB (B), FAB (C) and FEB (D) on the antibiotic activity of amikacin, gentamicin, benzylpenicillin and cefotaxime against the *Salmonella choleraesuis* (INCQS 00038) strain. Control (A). The numbers 1–4 express the significance of the association between fraction + antibiotic. 1: *p* < 0.05, 2: *p* < 0.01, 3: *p* < 0.001 e 4: *p* < 0.0001. Data were analyzed by ANOVA and Bonferroni’s test.

The analysis of modulating activity of the fractions of *M. flexuosa* by direct contact with antibiotics against bacterial strains showed that the fractions caused both synergistic and antagonistic effects, as can be observed in Figs. 4 and 5. The chloroform fraction exerted a synergistic effect in combination with gentamicin against *B. cereus* and *S. choleraesuis*, reducing the MIC of this antibiotic from 512 to 256 µg/mL. A similar effect was obtained with the association of this fraction with cefotaxime against *B. cereus*, in which the MIC of the antibiotic was reduced from 1,024 to 256 µg/mL. On the other hand, the fraction exerted an antagonistic effect when combined with benzylpenicillin against *B. cereus*, increasing the MIC of the penicillin from 21.3 µg/mL to 170.6 µg/mL.

In the tests with *S. choleraesuis,* the FCB reduced the MIC of Amikacin from 512 µg/mL to 170.6 µg/mL, whereas the FEB decreased it from 512 µg/mL to 341.3 µg/mL. In association with cefotaxime, against the same microorganism, all the fractions exerted the same synergistic effect, decreasing the MIC of the antibiotic from 1,024 µg/mL to 512 µg/mL. Finally, when associated with benzylpenicillin against this microorganism, only FAB and FEB potentiated the action of the antibiotic, reducing its MIC from 1,024 µg/mL to 426.6 and 512 µg/mL, respectively.

**Figure 6:**
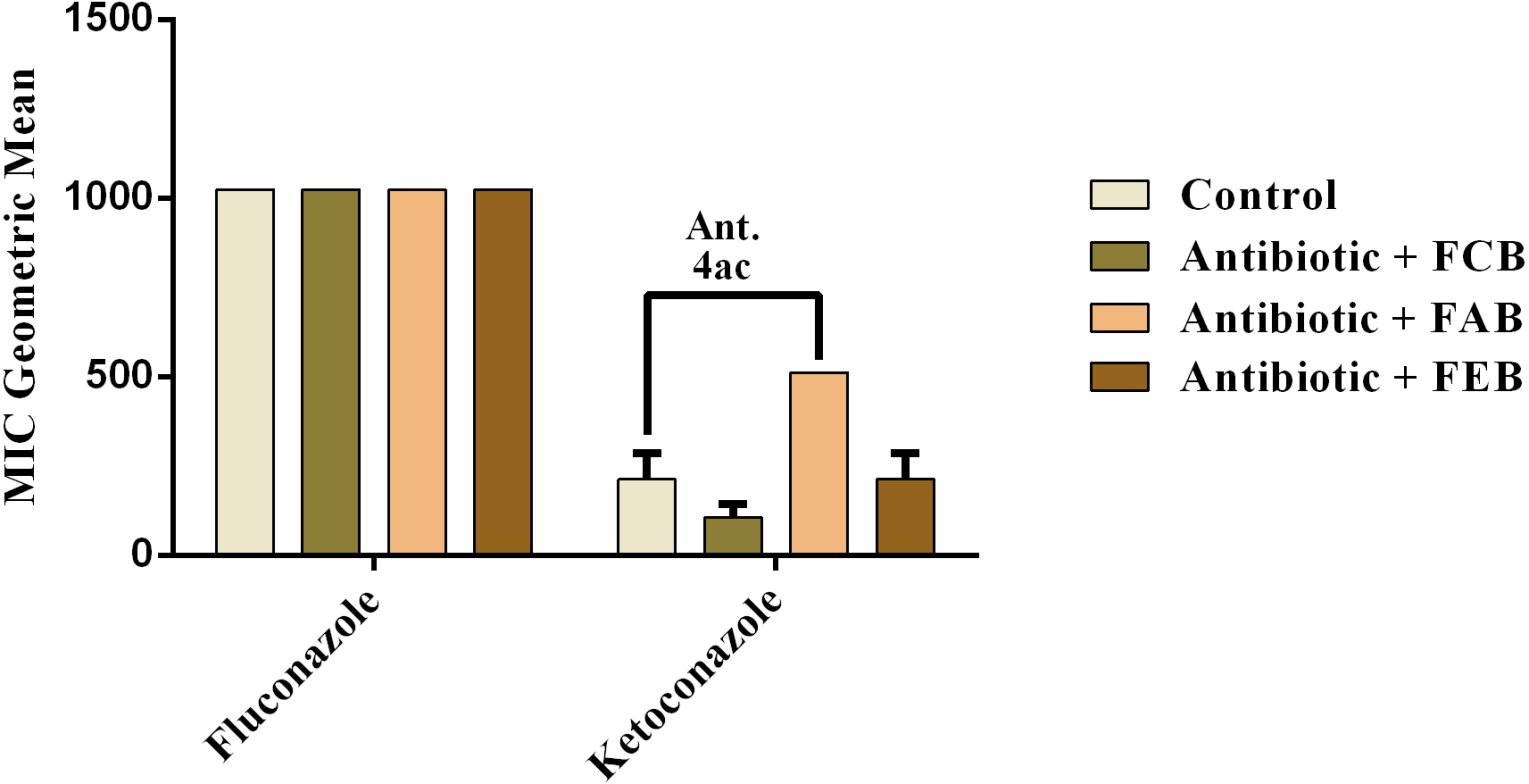
Modulating effect of FCB (B), FAB (C) and FEB (D) on the antibiotic activity of fluconazole and ketoconazole against the *Candida tropicalis* (INCQS 40042) strain. Control (A). The numbers 1–4 express the significance of the association between fraction + antibiotic. 1: *p* < 0.05, 2: *p* < 0.01, 3: *p* < 0.001 e 4: *p* < 0.0001. Data were analyzed by NOVA and Bonferroni’s test.

In the analysis of the antifungal activity modulation, the fractions did not present significant effects, when associated to the drugs against *C. albicans* and *C. krusei*. The only significant modulation was obtained from the association of the ethyl acetate fraction with on ketoconazole against *C. tropicalis*. This association presented an antagonistic effect, because the MIC of the drug was increased from 213.3 to 512 µg/mL, as shown in Fig. 6. These data indicate that, at the experimental conditions of the present work, the fruits of buriti might not be useful in the modulation of resistant fungal infections.

## Discussion

### Chemical analysis

The determination of the contents of phenolic compounds and flavonoids is relevant because the antioxidant activity of several plant extracts has been attributed to the presence of these compounds, especially flavonoids (Sousa et al., 2015). In addition, the structures of these compounds facilitate their action against several microorganisms as they form complexes with extracellular and soluble proteins, causing rupture of microbial membranes as well as enzymatic inhibition (Cowan, 1999; Cushnie & Lamb, 2005).

Flavonoids can be found in fruits, vegetables and beverages such as teas and wines (Nijveldt et al., 2001). These compounds present numerous biological activities against several diseases: inflammation, thrombosis, osteoporosis, diabetes, viral infections, liver diseases and ulcers, in addition to presenting vasodilatory effects (Tapas, Sakarkar & Kakde, 2008; Agrawal, 2011).

Structurally, polyphenols are defined as compounds having benzene rings with one or more hydroxyl substituents (Angelo & Jorge, 2007). Based on the chemical structures of the phenolic patterns used in this study (Fig. 2), it is justified that these substances are directly associated with activities such as complexation of metal ions, proteins and polysaccharides, as well as the sequestration of free radicals (Pereira & Cardoso, 2012).

These results of quantification of phenolic compounds and flavonoids by HPLC-DAD show that there are differences in the concentrations of the compounds in each fraction. In the present study, we suggest that differences in the phenolic content of the samples may be corelated with the polarity of the solvent, which influences extraction of a specific group of compounds (Najjaa et al., 2011; Milanez et al., 2018).

Orientin is a C-glycoside flavonoid that is obtained by the glycosylation of luteolin (Luteolin-8-C-glycoside). Previous studies reported the presence of this compounds in other species of the family Arecaceae (Hassanein et al., 2015; Heinrich, Dhanji & Casselman, 2011; Finco et al., 2012; Yao et al., 2012). This flavonoid is recognized for exhibiting biological activities such as antioxidant, anti-adipogenic, antithrombotic, anti-inflammatory, anti-platelet and protective against radiation (Devi et al., 2000; Kim et al., 2010; Praveena et al., 2014; Yoo et al., 2014; Lee & Bae, 2015).

Caffeic acid, also referred to as 3,4-dihydroxycinnamic acid, is among the metabolites of hydroxycinnamate and phenylpropanoids the most distributed in plant tissues (Fu et al., 2010). It is also found in other species of the Arecaceae family, where they are responsible for antimicrobial, antioxidant, anti-inflammatory and anti-angiogenic bioactivities (Chakraborty & Mitra, 2008; Beskow et al., 2014; Marques et al., 2016; Taleb et al., 2016).

### Antioxidant assays

According to our data, the fractions of buriti showed higher antioxidant capacity than other traditionally important fruits of the Arecaceae family, such as: açaí (*Euterpe oleracea* Mart.), Carnauba (*Copernicia prunifera* (Mill.) HE Moore) and juçara (*Euterpe edulis* Mart.), since literature data report that these plants presented levels of 15.1  ± 4.1 µM Trolox/g, 10.7 ± 0.2 µM Trolox/g and 78.3 ± 13.3 µM Trolox/g, respectively (Rufino et al., 2010).

A study by Almeida et al. (2011) reported the antioxidant activity of 11 fresh fruits from the Brazilian Northeast by the ABTS capture method, obtaining values of 15.73 ± 0.01 to 0.63 ± 0.01 µM of Trolox/g. The values obtained in the present study for buriti fractions were higher than the contents reported for those 11 fruits, which are considered good antioxidants.

Excessive iron accumulation can induce significant toxicity both by generation of reactive oxygen species and because of the lack iron excretion regulation in humans (Nurchi et al., 2016). Previous studies have shown the correlation between phenolic compounds and antioxidant activity, in addition to associating the chelating activity of natural products with their composition in the samples (Loizzo et al., 2012; Koolen et al., 2013; Cândido, Silva & Agostini-Costa, 2015; Tauchen et al., 2016). In fact, the structure of phenolic compounds, such as phenolic acids and flavonoids, is decisive for radical elimination and metal chelating activities. In addition, activity of these compounds depends on the number and position of hydroxyl groups in relation to the carboxyl functional group (Tapas, Sakarkar & Kakde, 2008; Garcia-Salas et al., 2010).

According to Loganayaki & Manian (2010), primary antioxidants prevent the onset and spread of oxidative chain reactions by neutralizing free radicals, and secondary antioxidants act to suppress oxidative damage by inhibiting radical formation. Thus, the fruit pulp fractions of *M. flexuosa* can act both as primary and secondary antioxidants because they exhibit antioxidant activity both by inhibiting free radicals and chelating iron ions.

### Antimicrobial assays

In the antibacterial assays, the fractions that had presented MICs ≥1,024 µg/mL against strains tested indicate low to moderate activities (Holetz et al., 2002). In fact, MICs ≥1,024 µg/mL present no clinically significant effects against a given bacteria, because concentrations above 1,000 µg/mL require a high dose of the natural product to achieve this concentration at plasma levels (Houghton et al., 2007). Regarding the antifungal activity, all strains presented MICs of 512 µg/mL, indicating that they present moderate antifungal activities (Matias et al., 2015).

Phenolic compounds are also recognized for their antimicrobial activities because they can interact with membrane proteins through hydrogen bonds involving the hydroxyl groups. This interaction can cause changes in membrane permeability that can lead to cell destruction (Tapas, Sakarkar & Kakde, 2008; Oussaid et al., 2017).

This study shows that the MFFs were more effectiveness against Gram-positive bacteria, corroborating with earlier reports that indicates that these microorganisms are more susceptible to the effects of plant extracts, because Gram-negative bacteria have a membrane that surrounds the cellular wall, which limits the diffusion and consequent effect of the compounds present in the extracts (Murari et al., 2008; Tian et al., 2009).

From these results, it is possible to correlate the modulating action of the fractions with their phenolic content, which is supported by studies that reported the action of these compounds as antibiotic modifiers. Lima et al. (2016) reported the drug modifying activity of phenolic acids, including caffeic acid, which potentiated the activity of antimicrobial agents against bacterial and fungal strains. Diniz-Silva et al. (2017) showed the potentiating activity of fruit flavonoids (in both aglycone and glycosylated forms) in association with norfloxacin against *Staphylococcus aureus*. According to the study, 3-O-glycoside flavonoids, such as hesperetin and quercetin, modulated the antibiotic resistance in this strain.

Aminoglycosides, in general, exhibit characteristic cellular toxicity as a function of their absorption into the intracellular environment, including: nephrotoxicity, ototoxicity and neuromuscular blockade (Oliveira, Cipullo & Burdmann, 2006). The combination of this class of antibiotics and natural products may be an alternative to reduce their side effects, because a significant reduction of the MIC also reduces the therapeutic dose (Figueredo et al., 2013).

Currently, the strategy used to overcome the bacterial resistance mechanism against beta-lactam antibiotics, is to associate this drugs with beta-lactamase inhibitors (Veras et al., 2017). The probability of resistance development during the treatment with antibiotics is significantly high, justifying the search for strategies to overcome these mechanisms. In this context, the development of efflux pump inhibitors that can be used as therapeutic adjuvant has been considered an attractive option (Tang, Apisarnthanarak & Hsu, 2014). Thus, due to the great pharmacological potential, natural products are important sources of compounds that can be useful in the management of infections caused by resistant microorganisms (Gibbons, 2004).

The presence of chitin contributes to the mechanical resistance of fungal cell walls, that are target of azoles, which act by interfering with ergosterol synthesis in the cell membrane of these microorganisms (Merzendorfer, 2006; Pfaller, 2012). Resistance to azoles in *Candida* species can occur by several mechanisms, such as presence of efflux pumps, point mutations that can alter the synthesis and cause overexpression of ergosterol and mutations that impair the membrane rupture, such as by-pass pathways (Pfaller, 2012). Therefore, these mechanisms might be associated with the results obtained by the present study.

Despite advances in prevention, diagnosis and therapy, invasive fungal infections still cause significant mortality and morbidity in immunocompromised patients and thus, antifungal resistance remains a worrying condition (Kanafani & Perfect, 2008). Currently, the therapy of these infections is limited to three main antifungal classes (azoles, echinocandins and polyenes), which present significant toxic effects, highlighting the importance of the search for new antifungal agents (Morais-Braga et al., 2016).

## Conclusion

The data obtained in this work demonstrated that the chloroform, ethyl acetate and ethanolic fractions obtained from the fruit pulp of *M. flexuosa* presented moderate antioxidant activity by acting both as primary and secondary antioxidants. The fractions presented antimicrobial and antibiotic potentiating activities, being the first record of modulating effect of fractions of this species against the studied microbial strains, but failed in modulating the activity of antifungal drugs, indicating that this plant has the potential to be used in the development of therapeutic alternatives against resistant bacteria.

The fractions presented a constitution rich in phenolic compounds, especially flavonoids, which may be responsible for their pharmacological properties *in vitro*. Therefore, the results obtained in this work contributed to elucidate the chemical and biological properties of *M. flexuosa* as a potential source of new bioactive compounds.

##  Supplemental Information

10.7717/peerj.5991/supp-1 Supplemental Information 1 Chelation raw data

10.7717/peerj.5991/supp-2 Supplemental Information 2 Modulation raw data

## Additional Information and Declarations

### Field Study Permissions

The following information was supplied relating to field study approvals (i.e., approving body and any reference numbers): Field experiments were approved by the Biodiversity Information and Authorization System (SISBIO) of the Chico Mendes Institute for Biodiversity Conservation (ICMBio) (project number: 61003-1).

### Data Availability

The following information was supplied regarding data availability: The raw data are provided in the Supplemental Files.
